# Gint4.T-Modified DNA Tetrahedrons Loaded with Doxorubicin Inhibits Glioma Cell Proliferation by Targeting PDGFRβ

**DOI:** 10.1186/s11671-020-03377-y

**Published:** 2020-07-20

**Authors:** Feng Wang, Yanghao Zhou, Si Cheng, Jinhe Lou, Xiang Zhang, Qiuguang He, Ning Huang, Yuan Cheng

**Affiliations:** 1 Department of Neurosurgery, The Second Affiliated Hospital of Chongqing Medical University, Chongqing, 400010 China; 2 Department of Orthopedics, The Second Affiliated Hospital of Chongqing Medical University, Chongqing, 400010 China; 3 Department of Neurology, Chongqing General Hospital, Chongqing, 400013 China

**Keywords:** Glioma, Platelet-derived growth factor receptor β, Gint4.T, DNA tetrahedron, Nanostructures

## Abstract

Glioma is one of the deadliest intrinsic brain tumours due to its invasive growth. The effect of glioma treatment is poor because of the presence of the blood-brain barrier and blood tumour barrier and insufficient drug targeting. DNA tetrahedrons (TDN) show great potential for drug delivery and may be a novel therapeutic strategy for glioma. In this study, we used TDN to deliver doxorubicin (DOX) for the glioma therapy. Gint4.T, an aptamer that could recognize platelet-derived growth factor receptor β on tumour cell, was used to modify TDN (Apt-TDN) for targeted drug delivery. The TDN were self-assembled by one-step synthesis, which showed small size (10 nm) and negative charge. Fetal bovine serum test showed its stability as a drug delivery vehicle. Apt-TDN could be effectively taken up by U87MG cells. Compared with DOX and DOX@TDN (TDN loaded with DOX), the DOX@Apt-TDN (Gint4.T-modified TDN loaded with DOX) showed more early apoptosis rate, higher cell cycle arrest, and greater cytotoxicity towards U87MG cells. In conclusion, our findings indicated that DOX@Apt-TDN provides a novel therapy with promising clinical application for gliomas patients.

## Introduction

Glioma, a tumour derived from the neuroepithelium, is the most common intracranial malignancy. Nearly 1/3 of all brain tumours are gliomas, and approximately 4/5 of primary malignant brain tumours are gliomas [1–4]. Currently, the most effective treatment for glioma is surgical resection and postoperative concurrent chemoradiotherapy, but unfortunately, the prognosis for patients remains poor. Traditional chemotherapy for glioma does not show a good outcome due to poor tumour targeting, complications due to the blood-brain barrier (BBB) and blood tumour barrier (BTB), and insufficient drug targeting. The BBB is the most important factor that prevents the delivery of almost all macromolecules (including drugs and genes) to the brain parenchyma and their proper function. Drugs that cannot cross the BBB have to be administered at sufficiently high doses to achieve an effective treatment concentration in the area of interest. However, excess drugs can cause severe systemic side effects and undesired drug accumulation in unaffected tissues. Moreover, existing conventional anti-glioma drugs have insufficient targeting capabilities [3, 4]. Nanoparticles have emerged as the most promising drug-carrying tool. Because of their size advantage, nanoparticles can cross the BBB and exert an antitumour effect. Kafa et al. [5] designed chemically functionalized multi-walled carbon nanotubes (f-MWNTs) targeting ANG and confirmed their ability to cross the BBB through in vivo and in vitro experiments. However, these nanomaterials may be distributed to various organs throughout the body or even enter the central nervous system (CNS), where they may cause neurotoxicity [6].

DNA is an ideal material for nanostructure construction because its assembly can be precisely controlled by Watson-Crick base pairing [7]. To date, a number of two-dimensional (2D) and three-dimensional (3D) DNA nanostructures have been designed and demonstrated [8–10]. Tetrahedral DNA nanostructures (TDN) have attracted significant attention because of their biocompatibility, stability, abundant functionalized modification sites, and low immunogenicity [11–13]. Turberfield et al. synthesized DNA tetrahedral nanostructures with a high yield using a one-step synthesis method [14]. Walsh et al. found that a nucleic acid probe-containing DNA tetrahedron could enter mammalian cells without the need for transfection reagent [15]. Lee et al. demonstrated that self-assembled tetrahedral nanoparticles can be used for targeted siRNA delivery in vivo [16]. TDN have shown excellent application prospects in molecular diagnostics, molecular delivery, and targeted drug therapy. They are also widely used in research on tumours in various organs, such as breast cancer [17]. Similarly, TDN have also been used in the study of nervous system diseases. Tian et al. [18] used DNA tetrahedrons as a foundation and modified them with angiopep-2 (ANG) to successfully construct the nanoprobe ANG-TDN, which could target low-density lipoprotein receptor-related protein 1 (LRP-1) for targeted imaging. Research has shown that ANG-TDN can cross the BBB. Ma et al. [19] suggested that tetrahedral DNA nanostructures can enter neural stem cells (NSCs) without the need for transfection agents, where they promote neural stem cell migration, proliferation, and differentiatio n[20], and have great potential for neural tissue repair and regeneration. Therefore, we investigated the possibility of the use of TDN in glioma therapy and enhancing its targeting ability.

Platelet-derived growth factor receptor β (PDGFRβ) is an important member of the tyrosine protein kinase family that is involved in cellular proliferation, migration, and angiogenesis. Several studies have shown that PDGFβ is a promising target for antitumour therapies because of its role in angiogenesis [21, 22]. Aptamers are short, single-strand DNA or RNA oligonucleotides that are produced by the systematic evolution of ligands by exponential enrichment (SELEX) method. Aptamers are similar to antibodies, which have high affinity and specificity towards their targets [23]. Due to their unique properties, aptamers play an important role in the targeted delivery of chemotherapeutic agents, siRNAs, and drug-loaded nanoparticles. Gint4.T, an RNA aptamer that can specifically bind PDGFRβ, is also a PDGFRβ-specific antagonist [24]. Monaco et al. [25] suggested that Gint4.T aptamers can cross the blood-brain barrier (BBB) and specifically recognize PDGFRβ. Gint4.T-conjugated polymeric nanoparticles (PNPs) can be readily taken up by glioblastoma (GBM) cells. In this study, we report a novel drug-loaded system that combines the Gint4.T aptamer and TDN. The Gint4.T-modified TDN (Apt-TDN) loaded with DOX (DOX@Apt-TDN) exhibited enhanced specific cellular uptake and cytotoxicity against U87MG cells.

## Methods

### Materials

All DNA oligonucleotides and 2’F-Py RNA oligonucleotides were purchased from Sangon Biotech (Shanghai, China), and all oligonucleotide sequences are listed in Table 1. GelRed DNA gel stain solution was purchased from Sangon Biotech. Both foetal bovine serum (FBS) and Dulbecco’s modified Eagle’s medium (DMEM) were purchased from Thermo Fisher (New York, USA). Doxorubicin (DOX) was purchased from Mengbio Technology (Chongqing, China). U87MG cells were purchased from Shanghai Life Academy of Sciences Cell Library (Shanghai, China). DAPI was purchased from Zhongshan Golden Bridge Biotechnology (Beijing, China).

**Table 1:** Sequence of each single-strand DNA and RNA

ssDNA	DNA and RNA Sequence (5′→3′)
**S1**	ACATTCCTAAGTCTGAAACATTACAGCTTGCTACACGAGAAGAGCCGCCATAGTA
**S1’**	TTTTTTACATTCCTAAGTCTGAAACATTACAGCTTGCTACACGAGAAGAGCCGCCATAGTA
**S2**	TATCACCAGGCAGTTGACAGTGTAGCAAGCTGTAATAGATGCGAGGGTCCAATAC
**S3**	TCAACTGCCTGGTGATAAAACGACACTACGTGGGAATCTACTATGGCGGCTCTTC
**S4**	TTCAGACTTAGGAATGTGCTTCCCACGTAGTGTCGTTTGTATTGGACCCTCGCAT
**cy3-S2**	cy3-TATCACCAGGCAGTTGACAGTGTAGCAAGCTGTAATAGATGCGAGGGTCCAATAC
**Gint4.T**	UGUCGUGGGGCAUCGAGUAAAUGCAAUUCGACAAAAAAA

### Preparation of DNA Nanostructures

To assemble the DNA tetrahedron (Table 1), 2 μL of each oligonucleotide (S1, S2, S3, and S4) was added to 42 μL of TM buffer (10 mM Tris-HCl, 5 mM MgCl_2_, pH = 8). The DNA solution was then heated to 95 °C for 5 min and subsequently cooled to 4 °C for 2 min using a Bio-Rad PCR machine (California, USA) [26, 27]. The final concentration of TDN was 2 μM. TDN’ was prepared in the same manner except S1 was replaced by S1’. To synthesize the Apt-TDN, the Gint4.T aptamer was added to TDN at an equal molar ratio, and the mixture was incubated at 37 °C for 60 min. Before synthesis, the aptamer was subjected to a short denaturation-renaturation step (85 °C for 5 min, rapid cooling over 2 min and subsequently warming to 37 °C over 10 min) [25].

### Agarose Gel Electrophoresis

An agarose gel (3%) was run in 0.5× TEB buffer at 100 V for 30 min. The temperature of the electrophoresis apparatus was maintained at 0 °C by placing the apparatus in an ice bath. Before electrophoresis, GelRed was added to the agarose gel to stain the DNA strands. When the process was finished, a Bio-Rad fluorescence scanner (California, USA) was used to capture an image of the gel.

### Dynamic Light Scattering

A Malvern Zetasizer ZS90 (Malvern, UK) was used to measure the hydrodynamic size and zeta potential of the TDN. A total of 1 mL of the TDN (100 nM) was subjected to dynamic light scattering (DLS) analysis.

### Atomic Force Microscopy Imaging

The TDN were diluted to 100 nM with TM buffer (Tris-HCl buffer containing MgCl_2_). Then, 10 μL of each TDN sample was added to freshly cleaved mica and incubated for 10 min. The samples were subsequently imaged on an atomic force microscopy (AFM) instrument in AC mode (Agilent 5500, USA).

### Measurement of the Drug-Loading Capacity of the Prepared TDN

Doxorubicin was dissolved in deionized water to make a 500-μM storage solution. Doxorubicin at different concentrations (1 to 20 μM) was mixed with TDN (100 nM) or Apt-TDN (100 nM) for 6 h at room temperature (24–26 °C). The mixed solutions were then centrifuged at 12,000×*g* for 10 min to obtain the drug-loaded TDN. Then, 50 μL of the supernatants were removed and mixed with PBS at a 1:1 ratio. A Varioskan LUX microplate reader (California, USA) was used to measure the fluorescence intensity of doxorubicin (*λ*_ex_ = 480 nm and *λ*_em_ = 590 nm) to determine the amount of doxorubicin in the supernatants [28]. The concentration of doxorubicin loaded in the TDN was calculated by the standard curve and fluorescence intensity. We also mixed doxorubicin with the TDN at increasing molar ratios.

### Serum Stability of the TDN In Vitro

The TDN were mixed with complete medium and incubated at 37 °C for 0, 2, 4, 6, 8, 10, 12, or 24 h. The TDN solutions were mixed with FBS at a 1:1 ratio and incubated at 37 °C for 1, 3, 5, or 7 h. After incubation, the mixtures were run on a 3% agarose gel.

### Cytotoxicity of the TDN In Vitro

To determine the cytotoxicity of the TDN, U87MG cells at a concentration of 1 × 10^4^ cells/well were seeded onto a 96-well plate. The cell culture medium was removed, and fresh media containing 0–500 nM TDN were added and incubated for another 24 h and 48 h after overnight incubation. Then, 10 μL of CCK-8 solution was added to each well, and the mixture was incubated for 1 h. The absorbance at 450 nm was then measured using a microplate reader.

### Fluorescence Imaging

Cellular uptake of DOX and TDN was studied by fluorescence microscopy (Olympus, Tokyo, Japan). U87MG cells were seeded on coverslips in 24-well plates with medium containing 10% heat-inactivated foetal bovine serum and 1% penicillin and streptomycin and grown for at least 1 day at 37 °C in a humidified atmosphere with 5% CO_2_ until the cells reached at least 75% confluence. After incubation, the culture media were removed. Complete media containing 100 nM Cy3-TDN and Cy3-Apt-TDN were added and incubated for 3 h. TDN and Apt-TDN were labelled with Cy3 to detect the intercellular uptake of nanoparticles. To assess the cellular uptake of DOX, DOX (DOX 2 μM), DOX@TDN (DOX 2 μM), and DOX@Apt-TDN (DOX 2 μM) were added to U87MG cells and incubated for 3 h. After 3 h of treatment, the cells were fixed with 4% paraformaldehyde for 20 min in the dark and subsequently stained with 4′,6-diamidino-2-phenylindole (DAPI) for 5 min. The cells were washed with PBS three times and observed under a fluorescence microscope.

### Flow Cytometry

A total of 1 × 10^6^ U87MG cells were implanted into 6-well plates. After overnight incubation, the culture media were removed, and media supplemented with 100 nM Cy3-TDN, 100 nM Cy3-Apt-TDN, or 100 nM Cy3-Apt-TDN + 1 μM free Apt were added and incubated for 3 h. Then, the cells were fixed with 4% paraformaldehyde for 20 min, and flow cytometry was used to analyse the percentages of Cy-3-positive cells.

### Cell Cycle and Apoptosis

After treatment with DOX, DOX@TDN, or DOX@Apt-TDN for 24 h, 5 × 10^5^ cells were collected and fixed in 75% ice-cold ethanol overnight. Then, the cells were incubated with RNase and propidium iodide for 30 min at 37 °C in the dark. The cell cycle was investigated by flow cytometry. In addition, after the different treatments, the cells were stained with Annexin V-FITC/DAPI, and early apoptosis was explored.

### CCK-8 Assays

To determine cell viability, U87MG cells (5 × 10^3^) were seeded onto 96-well plates with 100 μL of medium and cultured overnight at 37 °C under an atmosphere containing 5% CO_2_. The medium was subsequently removed, and fresh medium containing DOX, DOX-TDN, or DOX-Apt-TDN was added. After 24 h of incubation, 10 μL of CCK-8 solution was added, and the cells were cultured for another 1 h. A microplate reader was used to measure the absorbance at 450 nm.

### Statistical Analysis

All experiments in this study were performed in triplicate, and all data are presented as the mean value with its standard deviation (mean ± SD). Statistical analysis was performed using the SPSS 24.0 programme (IBM, USA). Significant differences were determined using Student’s *t* test, with *P* < 0.05 indicating significant differences between groups.

## Results

### Synthesis and Characterization of the TDN and Apt-TDN

**Fig. 1:**
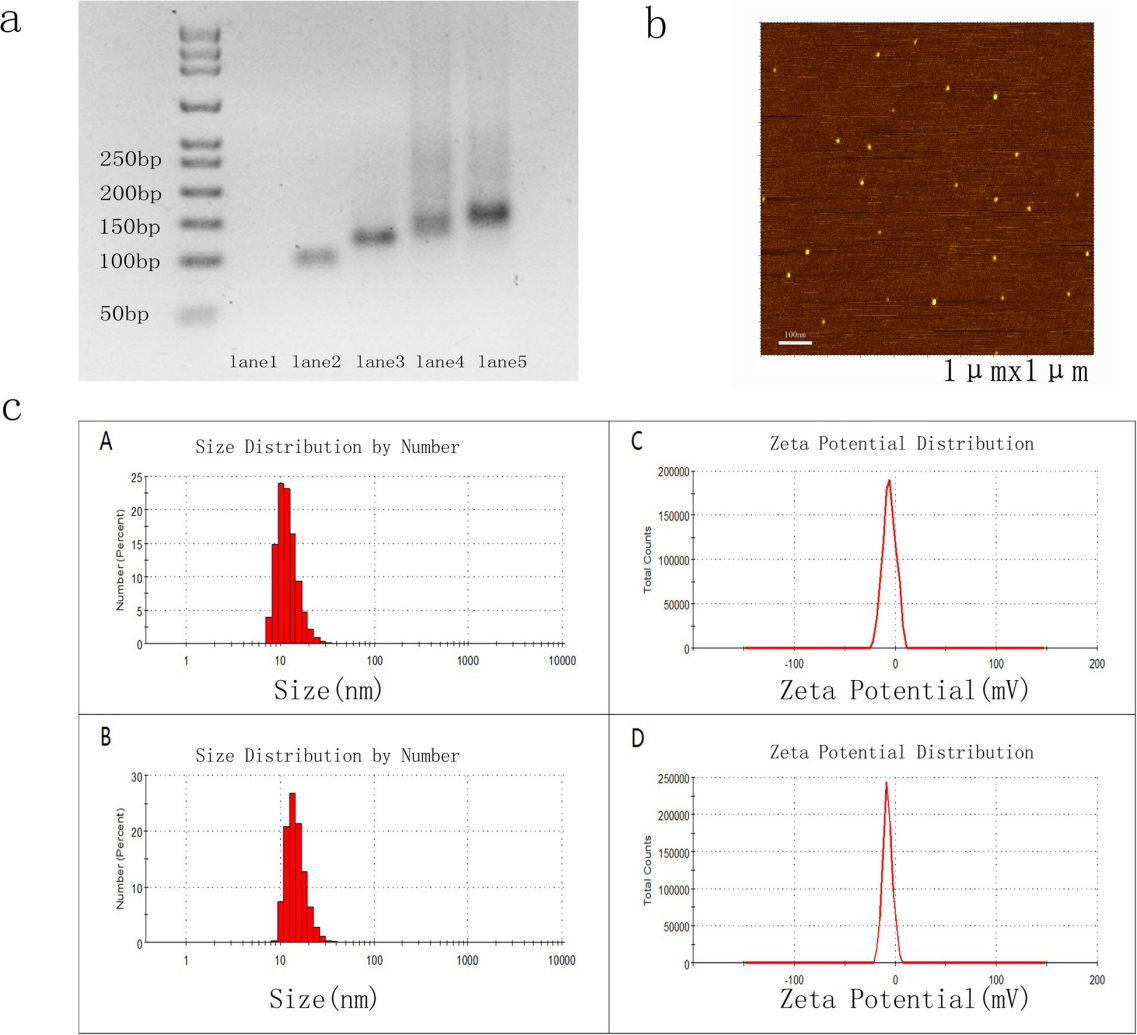
**a** Synthesis of the DNA tetrahedron and Gint4.T-TDN. Lane 1: S1; lane 2: S1+S2; lane 3: S1+S2+S3; lane 4: S1+S2+S3+S4 (TDN); lane 5: TDN mixed with Apt-tail (Gint4.T); Apt-TDN. Lane 1 was not visible because nucleic acid dyes cannot properly stain single-strand DNA. **b** AFM images showed that the heights of the TDN and Apt-TDN were ~ 2 nm. **c** Determination of the particle size and zeta potential of the TDN and Apt-TDN by dynamic light scattering (DLS). The average particle sizes of the TDN and Apt-TDN were 10.10 nm (A) and 13.54 nm (B), respectively. The average zeta potentials of the TDN and Apt-TDN were − 5.69 mV (C) and − 7.3 mV (D), respectively

The TDN was self-assembled from four oligonucleotides (Table 1) via single-step synthesis as previously reported [18, 29]. The tumour-targeting aptamer Gint4.T was used to modify TDN via Watson-Crick base pairing. The DNA tetrahedron contains four faces, with each face formed by one oligonucleotide. Thus, four oligonucleotides hybridized with each other to form a DNA tetrahedron (Fig. 1a). Gel electrophoresis analysis showed a single prominent band in lanes 4 and 5, suggesting that the TDN and Apt-TDN were successfully constructed. The mobility of the Apt-TDN was decreased compared to that of the TDN, suggesting that the Gint4.T aptamer successfully modified the TDN.

The sizes of the TDN and Apt-TDN were determined by DLS and AFM. The TDN and Apt-TDN showed particle sizes of 10.1 nm and 13.5 nm, respectively, reflecting the addition of the Gint4.T ligand (Fig. 1c (A), (B)). Because the hydrodynamic diameter includes water molecules, the particles were larger than their theoretical sizes. The heights of both the TDN and Apt-TDN determined by AFM images were ~ 2 nm (Fig. 1b), indicating that aptamer modification did not change the 3D structure. The average zeta potentials of the TDN and Apt-TDN were − 5.69 mV (C) and − 7.3 mV (D), respectively (Fig. 1c (C) (D)). Based on these parameters, we concluded that the TDN and Apt-TDN had successfully been assembled.

### Stability and Cytotoxicity of the TDN In Vitro

**Fig. 2:**
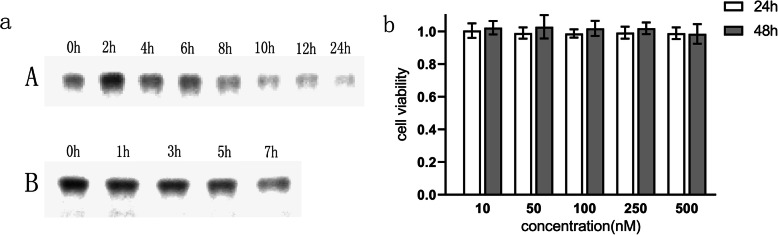
**a** Gel electrophoresis showed that the TDN remained stable for 24 h in complete medium at 37 °C (A); the TDN remained stable for 7 h when the concentration of foetal bovine serum was increased to 50% (B). **b** U87MG cells were co-cultured with the TDN at different concentrations (10–500 nM) for 24 h and 48 h. The CCK-8 assay showed that the activity of the U87MG cells was not affected, which indicated the biosafety of the TDN

Gel electrophoresis analysis showed that the TDN remained intact when incubated in complete medium for 24 h at 37 °C (Fig. 2a (A)). Furthermore, when the concentration of foetal bovine serum was increased to 50%, the TDN remained stable for at least 7 h (Fig. 2a (B)), which is consistent with previous reports [18, 26]. To determine the cytotoxicity of the nanostructure, the CCK-8 assay was used to assess the cell viability of U87MG cells after treatment with the TDN at a number of concentrations. As shown in Fig. 2b, no significant cytotoxicity was observed in U87MG cells treated with TDN at 0–500 nM for 24 h and 48 h. Hence, DNA nanoparticles can be used as stable and biosafe carriers for drug delivery.

### Drug-Loading Capacity of the TDN and Apt-TDN

**Fig. 3:**
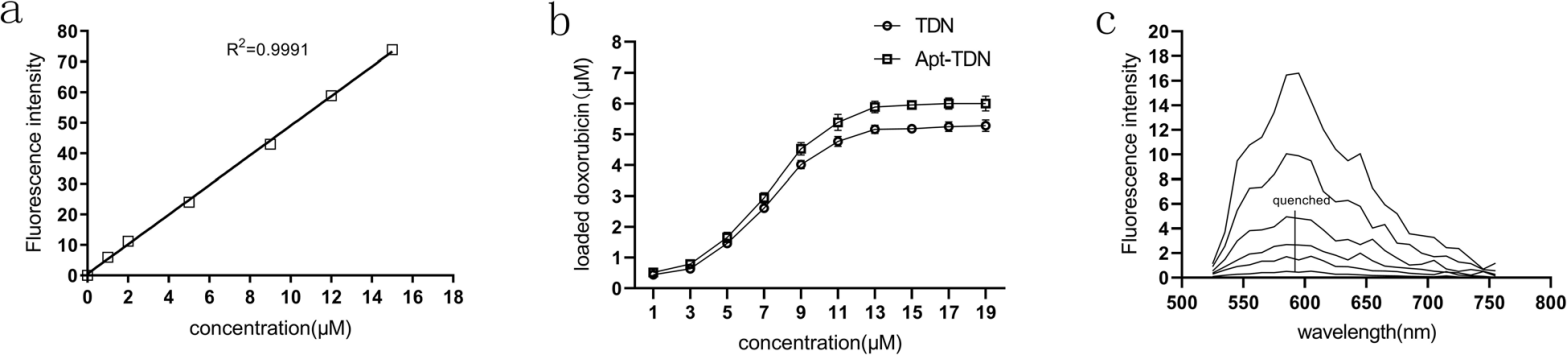
**a** A standard curve of DOX concentrations in PBS buffer; λex = 480 nm and λem = 590 nm. The amount of DOX carried by the TDN and Apt-TDN. **b** DOX intercalated into the double-strand DNA of the TDN and Apt-TDN. When the DOX concentration reached 14 μM and the intercalated DOX concentration in the TDN and Apt-TDN peaked at 5.5 μM and 6.0 μM, respectively, a single DNA tetrahedron could carry 55 Dox molecules, while a single aptamer-modified DNA tetrahedron carried 60 DOX molecules. **c** Fluorescence spectra of DOX in the supernatant. Doxorubicin was mixed with the TDN at increasing molar ratios (0, 0.0005, 0.001, 0.005, 0.01, and 0.05 from top to bottom). When the molar ratio was 1:20, the fluorescence was quenched

Doxorubicin is a broad-spectrum chemotherapeutic drug that can intercalate into DNA double strands. We calculated a standard doxorubicin curve (Fig. 3a) and then investigated the intercalation of doxorubicin into the TDN. The amount of intercalated doxorubicin in the TDN and Apt-TDN gradually increased with increasing doxorubicin concentration. When the doxorubicin concentration was 14 μM, the amount of doxorubicin intercalated in the TDN and Apt-TDN peaked at 5.5 μM and 6.0 μM, respectively, and subsequently plateaued (Fig. 3b), indicating that the DNA strands were fully occupied. Meanwhile, we mixed doxorubicin with the TDN at increasing molar ratios. The fluorescence spectrum of doxorubicin was scanned for analysis. As shown in Fig. 3c, the fluorescence spectrum of doxorubicin was quenched with doxorubicin at a molar ratio of 0.05. Based on these findings, we concluded that approximately 55 molecules of doxorubicin were contained within a single TDN, while 60 molecules were contained within a single Apt-TDN.

### Targeted Cellular Uptake of the Apt-TDN

**Fig. 4:**
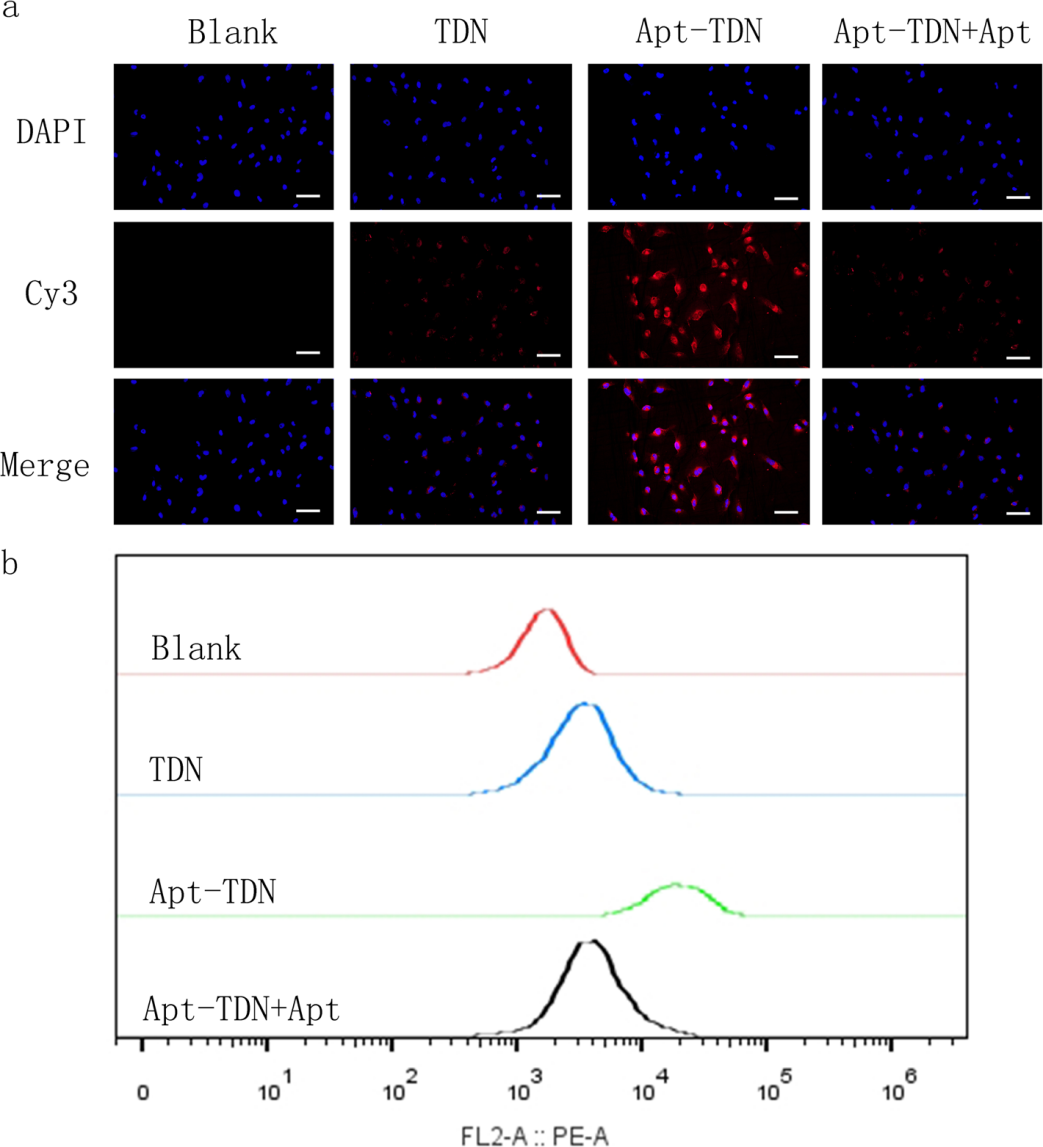
**a** U87MG cell uptake of the TDN and Apt-TDN (TDN-Gint4.T). The TDN entered U87MG cells directly without transfection agents, and the uptake of Apt-TDN (linked to the aptamer Gint4.T) was significantly increased and competitively inhibited by free Apt (Gint4.T), indicating that the aptamer Gint4.T plays a significant role in cellular targeting. The scale bar represents 50 μm. **b** Flow cytometry curves show the intracellular uptake of the TDN, Apt-TDN, and Apt-TDN+Apt after incubation for 3 h

DNA is a negatively charged macromolecule, which makes its entry into the negatively charged cell membrane difficult. Typically, individual DNA molecules must access cells with the help of transfection reagent. Here, we labelled TDN and Apt-TDN with Cy3 to monitor intracellular uptake of the nanoparticles. After incubation with U87MG cells for 3 h, red Cy3 fluorescence signal emerged in the cellular cytoplasm, indicating that the TDN bound the cytomembrane and were taken up into the cell without the help of transfection agents (Fig. 4a). The Apt-TDN showed higher red fluorescence, which suggests that the presence of the Gint4.T aptamer significantly increased DNA tetrahedron uptake by U87MG cells. However, when free aptamer was added, the Cy3 fluorescence decreased to the level observed for the TDN alone. Due to competitive inhibition by the free aptamer, we infer that the aptamer on the TDN could not facilitate uptake of the TDN. Based on this competitive inhibition, we proved that the Apt-TDN can target U87MG cells. Flow cytometry further proved that the percentage of Cy3-positive U87MG cells was higher in the Apt-TDN group than in the TDN group. Free Apt decreased the percentage of Cy3-positive U87MG cells in the Apt-TDN group (Fig. 4b).

### Cellular Uptake of the DOX@TDN and DOX@Apt-TDN

**Fig. 5:**
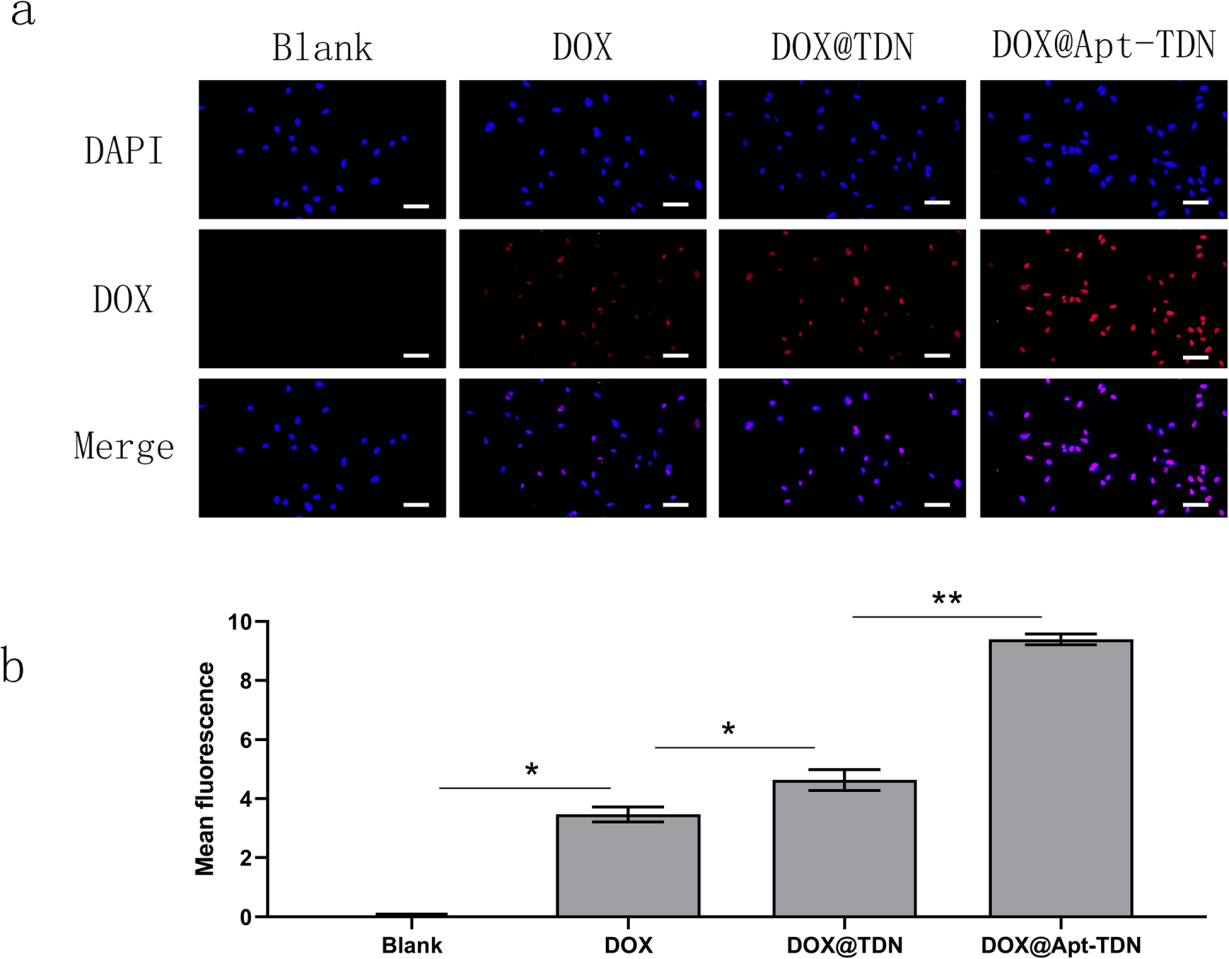
**a** Cellular uptake of DOX, the DOX@TDN, and the DOX@Apt-TDN. Modified with the aptamer Gint4.T, the Apt-TDN could deliver more doxorubicin to U87MG cells than the TDN. In addition, the TDN could carry more drug to cells than the drug alone. The scale bars indicate 50 μm. **b** Semi-quantitative analysis of the fluorescence intensity of doxorubicin with PBS, DOX, DOX@TDN, and DOX@Apt-TDN treatment (compared to blank: *p < 0.05, **p < 0.01)

We utilized the characteristic fluorescence spectrum of doxorubicin to assess drug uptake efficiency. After 3 h of treatment, intracellular doxorubicin was imaged by fluorescence microscopy (Fig. 5a). Free doxorubicin could enter U87MG cells and was located in the nucleus. With the addition of the DOX@TDN, the fluorescence was higher than that of free doxorubicin. This result suggested that DNA nanoparticles enhanced the cellular uptake of doxorubicin. When DOX@Apt-TDN was added, the red signal in the nucleus was even higher than that of cells to which DOX@TDN had been added. Semi-quantitative analysis of the intracellular uptake of DOX further confirmed that the Apt-TDN facilitated a more than twofold increase in intracellular DOX uptake compared with that for the single drug. We infer that this is due to Gin4.T-specific binding to receptors, following which more nanoparticles can enter the cell. After digestion in lysosomes, doxorubicin can be released into the cytoplasm and further functions in the nucleus.

### Cytotoxicity of DOX, the DOX@TDN, and the DOX@Apt-TDN

**Fig. 6:**
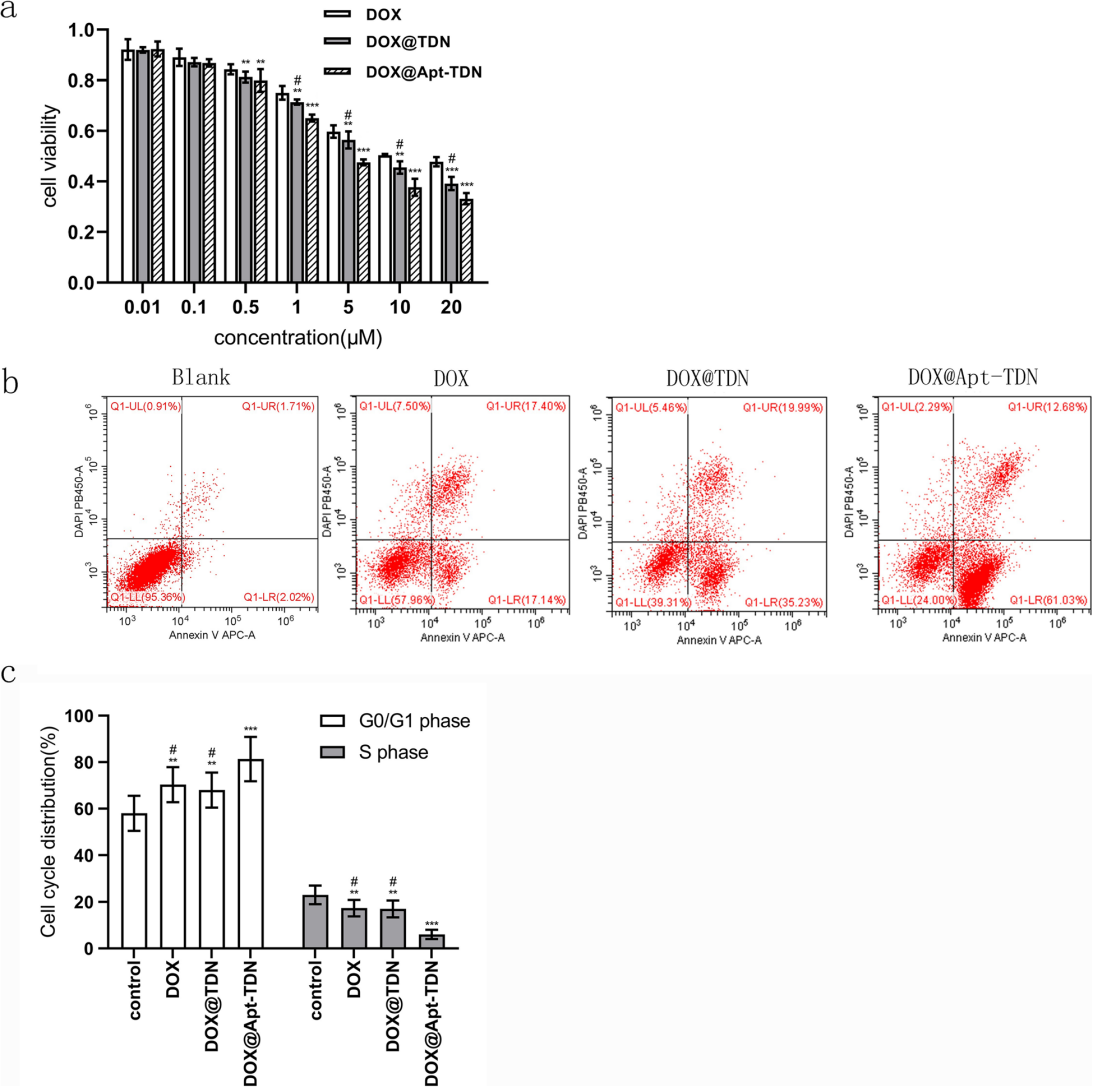
**a** Cytotoxicity of DOX, the DOX@TDN, and the DOX@Apt-TDN at various concentrations. The inhibition rate of the U87MG cells was significantly increased with increasing DOX concentration, but the DOX@TDN and DOX@Apt-TDN groups exhibited significantly increased cytotoxicity compared with the DOX group. The cell inhibition rate of the DOX@Apt-TDN group was also significantly higher than that of the DOX@TDN group (compared to DOX, **p < 0.05; compared to DOX, ***p < 0.01; compared to DOX@Apt-TDN, ^#^*p* < 0.05). **b** Apoptosis of U87MG cells after incubation with PBS, DOX, the DOX@TDN, and the DOX@Apt-TDN for 24 h. **c** Flow cytometry histograms of the U87MG cell cycle after incubation with PBS, DOX, the DOX@TDN, and the DOX@Apt-TDN for 24 h (compared to control, **p < 0.05; compared to control, ***p < 0.01; compared to DOX@Apt-TDN, ^#^*p* < 0.01)

For the cytotoxicity study, three groups of U87MG cells were treated with different concentrations of doxorubicin (Fig. 6a). The IC_50_ values for doxorubicin were 13.39 μM for DOX treatment, 7.826 μM for DOX@TDN treatment, and 4.205 μM for DOX@Apt-TDN treatment. Among the three treatments, the DOX@Apt-TDN showed the highest cytotoxicity at 24 h, which indicated the specificity of the Apt-TDN for U87MG cells. After 24 h, cells in the DOX, DOX@TDN, and DOX@Apt-TDN groups were collected and used to explore early apoptosis. Our data demonstrated that the early apoptosis rate was higher in the DOX@Apt-TDN group than in the other two groups (Fig. 6b). Moreover, the proportion of cells at G0/G1 phase was increased in the DOX-Apt-TDN group compared with the DOX and DOX-TDN groups (*p* < 0.01), and the ratio of cells at S phase was decreased in the DOX-Apt-TDN group (*p* < 0.01) (Fig. 6c). The percentage of cells at G2 phase was not changed (data not shown).

## Discussion

Whether via in vitro or in vivo experiments, the stability of a drug and its carrier must be determined. After assembly of the TDN, its stability was first determined in vitro. This study showed that the 3D structure of DNA nanoparticles can improve their stability in serum by inhibiting enzyme binding. The biological safety of the relevant nanostructure is the most important requirement for its application. No significant cytotoxicity was observed in U87MG cells co-cultured with various concentrations of the TDN for 24 h or 48 h. None of the DNA sequences used in this study encodes any genetic information, and no side effects were reported in the cytotoxicity test. Therefore, the TDN can serve as a safe and stable drug carrier.

The targeting efficiency of an aptamer-modified nanostructure is crucial for selective drug delivery to cancer cells. Unlike antibodies, aptamers are chemically stable, inexpensive, and can be mass produced. Furthermore, unlike other materials, aptamers can easily bind a DNA tetrahedron using the principle of base complementary. Thus, the combination of aptamers and DNA tetrahedrons lays the foundation for targeted drug delivery and next-generation treatments. Our results showed that the TDN could enter cells without transfection agents, which is similar to the results of Walsh et al. and Ma et al. [15, 19]. Compared with that of the TDN, uptake of the Apt-TDN by U87MG cells was significantly increased. After the addition of free aptamer, this increase disappeared. This result suggests that Gint4.T is a PDGFRβ-specific antagonist [25]. Our study demonstrated that the Gint4.T aptamer could target U87MG cells due to the high expression of PDGGRβ on the surface of glioma cells. Camorani et al. [24] also showed that the Gint4.T aptamer could target tumour cells by specifically interacting with the extracellular domain of PDGFRβ. The advantages of Gint4.T aptamer targeting have been demonstrated to some extent via in vitro studies, but further in vivo testing is required to confirm these findings. This study also confirmed that the DOX@Apt-TDN is more cytotoxic than DOX or the DOX@TDN, which likely originates from two factors. First, the Gint4.T aptamer can specifically bind PDGFR extracellular domains, blocking tumour cell proliferation and inhibiting tumour cell growth [24]. This is consistent with our experimental results. Compared with the control group, the U87MG cell cycle changes after treatment with DOX, DOX@TDN, and DOX@Apt-TDN increased significantly in the G0/G1 phase tumour cells, decreased in the S phase cells, and blocked tumour cells at G0/G1 phase, indicating that they can inhibit the cell cycle of U87MG cells and inhibit their proliferation. Compared with the DOX and DOX@TDN groups, the DOX@Apt-TDN group had a significantly stronger ability to inhibit the proliferation of U87MG cells. In addition, Gint4.T specifically binds tumour cells and enhances cell targeting of the DOX@Apt-TDN complex. Thus, we can increase the drug targeting efficiency and reduce the systemic administration dose of antitumour drugs to prevent their systemic side effects.

## Conclusions

Modification with Gint4.T increased the specificity and efficiency of TDN in glioma treatment by targeting the PDGFRβ that is expressed in large quantities on glioma cells. In addition, when loaded with Apt-TDN, it could significantly promote the anti-glioma effects of DOX. Therefore, DOX@Apt-TDN may serve as a promising therapeutic strategy against glioma for patients. The deficiency of this study is that it has only been verified in vitro. In the later study, we will further explore in animal models.

## Data Availability

The relevant data are included within the article.

## Abbreviations

BBB: Blood-brain barrier
BTB: Blood tumour barrier
TDN: Tetrahedral DNA nanostructures/DNA tetrahedrons
DOX: Doxorubicin
PDGFRβ: Platelet-derived growth factor receptor β
Apt-TDN: Gint4.T-modified TDN
DOX@ TDN: TDN loaded with DOX
DOX@Apt-TDN: Gint4.T-modified TDN loaded with DOX
CNS: Central nervous system
ANG: Angiopep-2
LRP-1: Low-density lipoprotein receptor-related protein 1
SELEX: Exponential enrichment
PNPs: Polymeric nanoparticles
GBM: Glioblastoma
FBS: Foetal bovine serum
DMEM: Dulbecco’s modified Eagle’s medium
DLS: Dynamic light scattering
AFM: Atomic force microscopy
CCK-8: Cell Counting Kit-8
Cy3: Sulfo-Cyanine3
DAPI: 4′,6-Diamidino-2-phenylindole
